# Partial regression of peripapillary myelinated nerve fibers after non-arteritic anterior ischemic optic neuropathy

**DOI:** 10.5935/0004-2749.2022-0237

**Published:** 2024-02-23

**Authors:** Francisco J Muñoz Negrete, Victor Aguado Casanova, Pablo Vicente Muñoz Ramón, Marta Gomez Mariscal, Teresa Salva Palomeque, Gema Rebolleda

**Affiliations:** 1 Ophthalmology Department, Hospital Universitario Ramón y Cajal, IRYCIS, Madrid, Spain; 2 Surgery Department, Universidad de Alcalá School of Medicine, Madrid, Spain

**Keywords:** Optic neuropathy, ischemic, Nerve fibers, myelinated, Optic nerve diseases, Tomography, optical coherence, Retinal neovascularization, Visual acuity, Humans, Case report, Neuropatia óptica isquêmica, Fibras nervosas mielinizadas, Doenças do nervo óptico, Tomografia de coerência óptica, Neovascularização retiniana, Acuidade visual, Humanos, Relato de casos

## Abstract

A 71-year-old woman presented a non-arteritic anterior ischemic optic neuropathy in an optic nerve with previously registered superonasal peripapillary myelinated nerve fibers. Her past medical history was significant for controlled systemic hypertension, hyperlipidemia, and diabetes mellitus. The physiologic cup was absent in both optic discs. Non-arteritic anterior ischemic optic neuropathy mainly affected the temporal and inferior sectors of the peripapillary retinal nerve fiber layer, as could be demonstrated by retinal nerve fiber layer optical coherence tomography and optic disc optical coherence tomography angiography. Unlike other published reports, just a slight regression of the myelinated nerve fibers was observed after 1 year of follow-up. This occurred because ischemia mainly affected the temporal and inferior peripapillary sectors, whereas myelinated nerve fibers were superonasal to the optic disc.

## Abstract (pt)

Uma mulher de 71 anos de idade apresentou neuropatia óptica isquêmica anterior não arterítica no nervo óptico com fibras nervosas peripapilares mielinizadas previamente registradas. Seu histórico médico foi significativo para hipertensão arterial sistêmica controlada, hiperlipidemia e diabetes mellitus. Em ambos os discos ópticos, a tacícula fisiológica esteve ausente. A neuropatia óptica isquêmica anterior não arterítica afetou principalmente os setores temporal e inferior da camada de fibras nervosas da retina peripapilar, como demonstrado pela tomografia de coerência óptica da camada de fibras nervosas da retina e pela angiotomografia de coerência óptica do disco óptico. Ao contrário de outros relatórios publicados, apenas uma ligeira regressão das fibras nervosas mielinizadas foi observada após um ano de acompanhamento. Isto pode ser explicado pelo fato da isquemia ter afetado principalmente os setores temporal e inferior peripapilares, enquanto as fibras nervosas de mielina eram nasal superior ao disco óptico.

## INTRODUCTION

Non-arteritic anterior ischemic optic neuropathy (NAION) is caused by acute ischemia, which affects the optic nerve head and subsequently results in retinal ganglion cell death. NAION is a common cause of optic neuropathy in patients aged >50 years. It typically presents as a sudden painless unilateral visual loss associated with relative afferent pupillary defect, disc edema, peripapillary hemorrhages, and altitudinal defects in the visual field (VF). Although the exact pathogenesis of NAION is unclear, transient hypoperfusion, small arterial occlusive disease, compartment syndrome, or occlusion of tributaries of the central retinal veins have been proposed as underlying mechanisms^(1)^.

Myelinated nerve fibers (MNF) are present in 0.57%-1% of the population and consist of retinal areas where nerve fibers have a myelin sheath. Ophthalmoscopically, MNF are described as gray-white sharply demarcated patches contiguous with the optic disc. In most cases, MNF are a congenital anomaly. During normal development, the lamina cribrosa (LC) may act as a barrier that prevents the access of oligodendrocytes and myelinization of the prelaminar fibers. Consequently, MNF occur when the LC fails to block the migration of oligodendrocytes. Some authors have postulated that MNF are oligodendrocytic choristomas rather than lesions secondary to the abnormal migration of oligodendrocytes^(2)^.

## CASE REPORT

A 71-year-old woman presented with a 1-week history of painless left eye visual loss. Previously, she had been followed up annually in our clinic because of a long-standing right iris nevus and left peripapillary MNF in the superior and nasal peripapillary regions. Her past medical history was significant for systemic hypertension (controlled with morning intake of amlodipine 5 mg and olmesartan 20 mg), hyperlipidemia, diabetes mellitus, and thyroiditis (levothyroxine 75 mg).

Her visual acuity (VA) was 1.0 in her right eye (RE) and 0.1 in her left eye (LE) (Snellen decimal notation). The pupillary examination revealed a left relative afferent pupillary defect. The anterior segment was unremarkable, except for her stable right iris nevus. The intraocular pressure (IOP) was 15 mmHg in both eyes. Funduscopic examination showed an edematous left optic disc (along with the MNF previously mentioned) and a normal right disc. The physiologic cup was absent in both optic discs.

Blood pressure and laboratory workup, including complete blood cell count, erythrocyte sedimentation rate, and C-reactive protein, were normal. Symptoms or signs of giant cell arteritis were not present. Consequently, the patient was diagnosed with NAION.

A 24-h ambulatory blood pressure monitoring (ABPM) was performed to rule out nocturnal hypotension. An episode of nocturnal diastolic hypotension (between 55 and 60 mmHg), which lasted for approximately an hour, was noticed.

**Figure 1:**
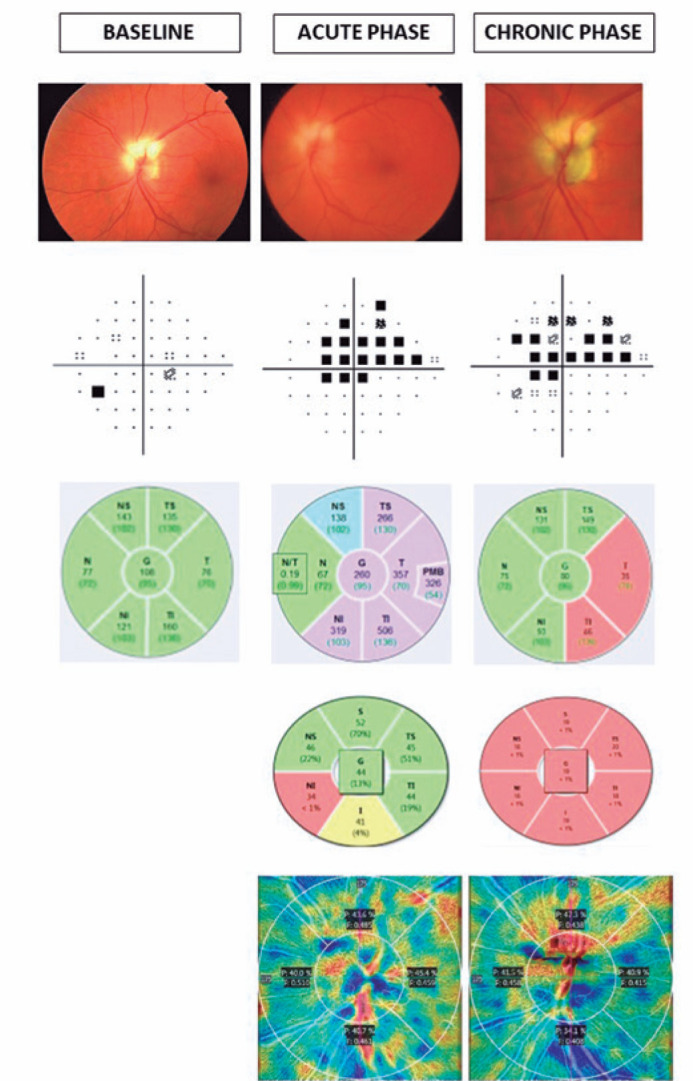
Fundus photograph, visual field, optical coherent tomography (retinal nerve fiber layer and ganglion cell layer thickness), and optical coherent tomography angiography of the left eye at baseline, non-arteritic anterior ischemic optic neuropathy diagnosis, and 12 months later. In the baseline visual field, a defect inferior to the blind spot corresponding to superior peripapillary myelinated nerve fibers is observed.

VF testing (standard Swedish Interactive Threshold Algorithm 24-2 strategy, Humphrey Field Analyzer; Carl Zeiss Meditec, Dublin, CA) showed a superior altitudinal defect. Spectralis (Heidelberg Engineering GmBH, Heidelberg, Germany) optical coherence tomography (OCT) of the retinal nerve fiber layer (RNFL) showed increased RNFL thickness in the inferior and temporal sectors, and ganglion cell layer (GCL) OCT showed a reduction in the nasal inferior sector. Optic disc OCT angiography (OCT-A; AngioPlex; CIRRUS, HDOCT-5000, 10.0, Carl Zeiss Meditec) showed a relative reduction in perfusion in the temporal (46.5% in RE and 45.4% in LE) and inferior (46.2% in RE and 40.7% in LE) sectors of the left eye (Figure 1).

**Figure 2:**
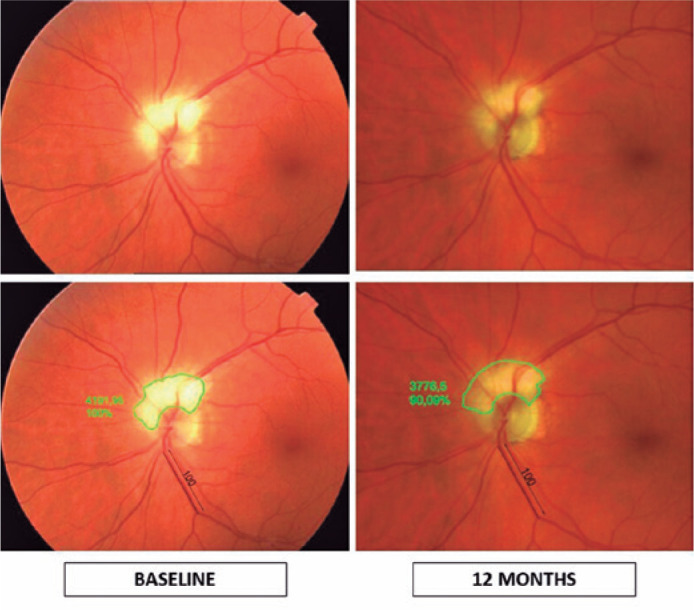
MNF area before the event and at 1-year follow-up. Top: retinography before the event and after 12 months of follow-up. Bottom: rough comparison of the MNF area before the event and after 12 months of follow-up. To compare the MNF area, the constant length of the superior retinal vein between two bifurcations was measured, and a random value (100) was given to this length in the baseline retinography. In this way, the retinography at 1-year follow-up was scaled, and the MNF area was calculated. A slight reduction in the MNF area of approximately 10% was observed. Measurements were made by a third blind technician using AutoCAD (2021), Autodesk Inc., Windows, Mill Valley, CA, USA.

Twelve months later, the VA was 0.15, and OCT showed a significant reduction of the temporal and inferotemporal sectors of the RNFL, corresponding with the superior altitudinal VF defect, and a significant reduction of all sectors of the GCL. OCT-A showed a greater reduction in the perfusion of the inferior sector (34.1%) of the left eye. The superior VF defect remained stable (Figure 1). A slight reduction in the MNF area of approximately 10% was observed (Figure 2).

## DISCUSSION

This patient had some typical systemic risk factors for NAION, i.e., age (71 years old), hypertension, diabetes mellitus, and hyperlipidemia. Although she was not taking antihypertensive drugs at bedtime, the ABPM showed an episode of nocturnal diastolic hypotension. In addition, the physiologic cup was absent at baseline (Figure 1). Therefore, NAION could be explained without MNF involvement.

After reviewing the literature on this topic, we found 17 cases of ischemic-related events in patients with peripapillary MNF reported between 1981 and 2021 (Table 1); thus, MNF may have played a role in these cases. The most frequently reported event was retinal neovascularization over the MNF area (11 of 17 patients, 64.71%) and usually presented as a recurrent vitreous hemorrhage that resolved after focal photocoagulation.

**Table 1:** Ischemia-related events reported in patients with peripapillary MNF between 1981 and 2021

Case	Year of publication	Authors	Ischemic event	Age	Sex	Risk factors	Clinical course
1	1981	Schachat^(4)^	NAION	45	M	no	MNF regressed 6 months after the event
2	1983	Minning^(6)^	RNV	47	M	no	Recurrent VH that resolved after focal photocoagulation and PRP
3	1987	Teich^(7)^	BRAO	54	M	T2DM, aortic stenosis, chronic renal failure	Both MNF and BRAO were inferotemporal to the optic disc MNF regressed after the event
4	1990	Kodama^(8)^	RNV, BRVO	50	F	controlled HBP	Recurrent VH that required vitrectomy and BRVO during follow-upBoth MNF and BRVO were superotemporal to the optic disc
5	1996	Leys^(9)^	RNV	15	M	no	Recurrent VH that resolved after focal photocoagulation
6	1996	Leys^(9)^	RNV	27	M	no	Mild VH that resolved spontaneously
7	1996	Leys^(9)^	RNV	30	F	no	Recurrent VH that required PRP and pars plana vitrectomy
8	1996	Leys^(9)^	RNV	43	F	no	Recurrent VH that resolved after focal photocoagulation MNF regressed after laser but arcuate scotoma was noticed
9	1996	Silvestri^(10)^	RNV	24	F	no	2-year history of recurrent VH that resolved after focal photocoagulation
10	1996	Silvestri^(10)^	RNV	48	M	no	Recurrent VH since childhood and amblyopia Treatment was not reported
11	1996	Silvestri^(10)^	RNV	32	F	no	Recurrent VH Treatment was not reported
12	2001	Munteanu^(11)^	CLRAO	44	F	no	MNF surrounded the optic nerve head 360^o^ MNF state after the event is not reported
13	2008	Berry-Brincat^(12)^	DNV	26	F	no	6-year history of recurrent VH that persisted despite the PRP
14	2008	Sellami^(13)^	RNV	31	F	no	VH that resolved after focal photocoagulation and cryotherapy
15	2013	Battaglia^(14)^	RNV	23	F	no	VH treated with three intravitreal injections of bevacizumab over a 24-month follow-up Cessation of fluorescein leakage was observed, but retinal neovascularizationdid not regress The patient had undergone focal photocoagulation 9 years earlier after recurrent VH
16	2013	Fard^(3)^	NAION	62	F	controlled HBP, sleep apnea	Sectoral regression of MNF 6 months after the event
17	2017	Karam^(15)^	CRAO	85	F	unknown	MNF regressed 3 months after the event

BRAO= branch retinal artery occlusion; BRVO= branch retinal vein occlusion; CLRAO= cilioretinal artery occlusion; CRAO= central retinal artery occlusion; DNV= disc neovascularization; F= female; HBP= high blood pressure; M= male; MNF= myelinated nerve fibers; PRP= panretinal photocoagulation; RNV= retinal neovascularization; T2DM= type 2 diabetes mellitus; VH= vitreous hemorrhage.

To the best of our knowledge, only two cases of NAION associated with peripapillary MNF have been published^(3,4)^. The increased thickness caused by the myelination of the RNFL in a predisposed optic disc could increase the typical crowded morphologic appea-rance that usually contributes to the development of NAION. Our patient had other systemic risk factors for NAION, similar to the case previously reported by Fard^(3)^. However, Schachat reported this association in a 45-year-old healthy patient^(4)^, which may indicate that MNF could be a risk factor.

Both cases had regression of the peripapillary MNF^(3,4)^. The regression of equatorial MNF after NAION was also described^(5)^. Conversely, in the present case, only a slight reduction in the MNF area was observed 12 months after the NAION episode (Figure 2). In this case, the ischemic episode was present in the temporal and inferior sectors of the peripapillary RNFL, as could be demonstrated by RNFL OCT and OCT-A, whereas the superior and nasal peripapillary areas that corresponded with the location of the MNF were less affected. This could explain why, unlike other cases, the MNF area only showed a slight regression.

MNF are generally benign lesions but coexist with ischemic events, such as NAION, in some patients. MNF could contribute to reducing the perfusion in a predisposed crowded optic disc. The MNF volume could be reduced when the ischemic event affects the corresponding area. Comparative studies are needed to confirm if MNF could be a new risk factor for NAION.

## References

[1] Miller NR, Arnold AC. (2015). Current concepts in the diagnosis, pathogenesis and management of
nonarteritic anterior ischaemic optic neuropathy. Eye (Lond).

[2] Rao R, Turkoglu EB, Say EA, Shields CL. (2019). Clinical features, imaging, and natural history of myelinated
retinal nerve fiber layer. Retina.

[3] Fard MA, Fakhree S. (2013). Sectoral loss of myelin and axons in anterior ischemic optic
neuropathy. Optom Vis Sci.

[4] Schachat AP, Miller NR. (1981). Atrophy of myelinated retinal nerve fibers after acute optic
neuropathy. Am J Ophthalmol.

[5] Raber FP, Werner JU, Kilani A, Lang GE. (2021). [Regression of myelinated retinal nerve fibres after anterior
ischemic optic neuropathy]. Klin Monbl Augenheilkd.

[6] Minning CA, Davidorf FH. (1983). Neovascularization associated with myelinated nerve fibers: a
case report. Ann Ophthalmol.

[7] Teich SA. (1987). Disappearance of myelinated retinal nerve fibers after a branch
retinal artery occlusion. Am J Ophthalmol.

[8] Kodama T, Hayasaka S, Setogawa T. (1990). Myelinated retinal nerve fibers: prevalence, location and effect
on visual acuity. Ophthalmologica.

[9] Leys AM, Leys MJ, Hooymans JM, Craandijk A, Malenfant M, van Germeersch D (1996). Myelinated nerve fibers and retinal vascular
abnormalities. Retina.

[10] Silvestri G, Sehmi K, Hamilton P. (1996). Retinal vascular abnormalities. A rare complication of myelinated nerve fibers? Retina.

[11] Munteanu M, Munteanu G, Giuri S. (2001). [Myelinated nerve fibers associated with cilioretinal artery
occlusion]. J Fr Ophtalmol.

[12] Berry-Brincat A, Shafquat S. (2008). Myelinated nerve fibres: a rare cause of recurrent vitreous
haemorrhage. Eye (Lond).

[13] Sellami D, Bouacida W, Maalej A, Ben Amor S, Châabouni M, Kamoun B, Feki J. (2008). [Retinal neovascularization with myelinated nerve
fibers]. J Fr Ophtalmol.

[14] Battaglia Parodi M, De Benedetto U, Vergallo S, Knutsson KA, Bandello F, Lanzetta P (2013). Intravitreal bevacizumab for retinal neovascularizations
associated with myelinated nerve fibers. J Ocul Pharmacol Ther.

[15] Karam E, Restrepo A, Assael S. (2017). Disappearance of myelinated retinal nerve fibers after central
retinal artery occlusion reveals Nettleship collaterals. Ophthalmology.

